# Understanding symptom profiles of depression with the PHQ-9 in a community sample using network analysis

**DOI:** 10.1192/j.eurpsy.2024.1756

**Published:** 2024-05-23

**Authors:** Catalina Núñez, Jaime Delgadillo, Michael Barkham, Alex Behn

**Affiliations:** 1 Millennium Institute for Depression and Personality Research (MIDAP), Santiago, Chile; 2 School of Psychology, Pontificia Universidad Católica de Chile, Santiago, Chile; 3 Clinical and Applied Psychology Unit, School of Psychology, University of Sheffield, Sheffield, UK

**Keywords:** depression heterogeneity, depression profiles, network analysis, symptoms of depression, depression

## Abstract

**Background:**

Depression is one of the most prevalent mental health conditions in the world. However, the heterogeneity of depression has presented obstacles for research concerning disease mechanisms, treatment indication, and personalization. The current study used network analysis to analyze and compare profiles of depressive symptoms present in community samples, considering the relationship between symptoms.

**Methods:**

Cross-sectional measures of depression using the Patient Health Questionnaire – 9 items (PHQ-9) were collected from community samples using data from participants scoring above a clinical threshold of ≥10 points (N = 2,023; 73.9% female; mean age 49.87, SD = 17.40). Data analysis followed three steps. First, a profiling algorithm was implemented to identify all possible symptom profiles by dichotomizing each PHQ-9 item. Second, the most prevalent symptom profiles were identified in the sample. Third, network analysis for the most prevalent symptom profiles was carried out to identify the centrality and covariance of symptoms.

**Results:**

Of 382 theoretically possible depression profiles, only 167 were present in the sample. Furthermore, 55.6% of the symptom profiles present in the sample were represented by only eight profiles. Network analysis showed that the network and symptoms’ relationship varied across the profiles.

**Conclusions:**

Findings indicate that the vast number of theoretical possible ways to meet the criteria for major depressive disorder (MDD) is significantly reduced in empirical samples and that the most common profiles of symptoms have different networks and connectivity patterns. Scientific and clinical consequences of these findings are discussed in the context of the limitations of this study.

## Introduction

### Why is depression a public health problem?

Depression is the most prevalent mental health problem in the world affecting 4.7% of the global population [1]. It has been classified as a public health problem due to its impact on quality of life, work productivity, and mortality risk [2]. Despite global efforts to understand and treat depression, its incidence has actually increased by 49% between 1990 and 2017 [3]. Currently, it is the third leading cause of disease burden and the single highest contributing factor to global disability [3, 4]. The impact of depression is not only felt by individuals, but also by their families and communities, who suffer a direct cost related to treatment and an indirect cost linked to an individual’s reduced functional capacity [5, 6]. Studies estimate that, when diagnosed, depression could cost $6,200 per person per year [7], while undiagnosed and untreated depression contributes to an even more significant burden of illness, increasing personal and societal costs [8]. Indeed, longer periods of undiagnosed and untreated depression lead to negative outcomes including poorer treatment response and lower remission rates [9], more severe cognitive impairment [10], and overall to poorer illness trajectories [11].

### What does heterogeneity of depression mean?

Ever since the publication of Diagnostic and Statistical Manual of Mental Disorders, third edition (DSM-III) forty years ago, the field of health care has mainly conceived depression as a homogeneous, distinct, and robust diagnostic category, outlined broadly in the polythetic system of the DSM as major depressive disorder (MDD) [12]. The DSM’s polythetic system masks a significant amount of syndromic heterogeneity, allowing for multiple combinations of symptoms to exist under the same diagnostic label [13]. As a consequence, the diagnostic criteria of MDD have led to the classification of people with only some or even no symptoms in common into the same broad category, ignoring the specific presentation of their symptoms and the interactions between specific symptoms [14–16].

### Consequences of heterogeneity of depression on treatment outcomes

Failure to consider the heterogeneity of depression has impacted the understanding of etiologic mechanisms and their physiologic correlates [17–20], and it has also limited the effectiveness of treatments [21]. Thus, it is highly important that research should consider the heterogeneity of MDD in order to better address etiologic processes and to implement smarter and personalized treatment strategies [16].

It is estimated that nearly 85% of people who recover from MDD suffer a second episode within 15 years and that each additional MDD episode increases the risk of relapse by 18% [22]. In addition, for 30% of patients diagnosed with MDD, symptoms do not remit despite varied treatment attempts [2], with sleep problems and fatigue being the most prevalent residual symptoms [23]. This highlights that patients will not respond similarly to different treatments for MDD [24, 25]. Even though clinicians typically adjust treatments to their specific patients, often guidelines recommend treatments’ packages that are delivered to the “average depressed patient” and insufficient research has considered what are the specific modifications that should be implemented to optimize a treatment for a particular subtype of depression. Thus, treatments may yield suboptimal effects, whereas parsing out heterogeneity of depression could enable the design of evidence-based personalization strategies for treatments, thus leading to possible improved patient outcomes.

### What are we missing by not looking at symptom-level heterogeneity?

Research has typically approached depression as a common cause for diverse symptoms and assumed that these symptoms are independent and have equal importance [14, 26, 27]. However, such a strategy has paid less attention to the interaction and mutual reinforcement between symptoms [28]. This is a problem, considering that researchers have been attempting to find associations between different symptoms of depression and distinct risk factors [29, 30], different gene polymorphisms [31], and different responses to treatment [32, 33]. Moreover, different symptoms have been associated with varying impacts on disability, with depressed mood and concentration problems being the most disabling symptoms [34]. This is consistent with research showing that patients who receive their optimal treatment (considering their specific symptoms and personal characteristics) had clinically significant improvements in depression [e.g., 21, 35, 36].

An examination of symptom-level heterogeneity of depression may also be crucial in improving our understanding of differential developmental pathways toward psychopathology from the perspective of equifinality and multifinality [37]. Indeed, by using a homogeneous conceptualization of depression, different pathways to illness may be masked, and thus, relevant opportunities for prevention and personalization lost. Heterogeneity research in depression can move the field toward a more person-centered approach that recognizes the relevance of different developmental pathways to illness that may be related to or represented by different profiles of depression [38].

Until now, depression has been studied through theoretical and empirical approaches that have supplied evidence to its heterogeneity, identifying profiles of symptoms that may help map out heterogeneity [2, 16]. However, although empirical research has found profiles related to the composition as well as severity of symptom profiles (e.g., [13, 39]), it does not consider the relation *between* symptoms within emerging profiles. Furthermore, studies show that not all theoretically possible profiles of symptoms are actually present in clinical samples [13, 39]. Still, these studies have focused on the presence and prevalence of different profiles, leaving aside how the symptoms are related to each other as an interrelated system. There are also studies that are focused on seeing the interaction between depressive symptoms using network analysis and other analytic strategies, but they usually analyze the depressive symptoms on total samples without considering different profiles and interrelated networks between profiles [40].

As a result, it is not known which symptoms are present in each profile, how they are related, or what the structure of the network of symptoms is like. In addition, we do not understand how the empirical frequency of theoretical profiles differs when considering community samples that include both help-seeking and non-help-seeking individuals. The current study is the first, to our knowledge, to examine the network structure and interactions between symptoms on different symptom profiles of depression.

## Methods

### Participants

The study used secondary data derived from three community studies with nationally representative samples: (1) the Chilean Longitudinal Social Survey (ELSOC), (2) the Longitudinal Study of Intercultural Relations (ELRI), and (3) the Social Protection Survey (EPS). These three studies were carried out between 2016 and 2020 and used multistage, stratified, and probabilistic sampling. The inclusion criteria for the sampling of these studies focused on female and male residents in urban areas, aged 18 to 99, and located in 13 different (blinded for review). All three studies used the Patient Health Questionnaire – 9 items (PHQ-9) to measure depression symptoms. Only participants with clinically significant depressive symptoms (PHQ-9 ≥ 10) were included in the present study. Of a total sample of 13,367 participants, 2,023 (15.13%) had a PHQ-9 score of 10 or above.

### Measures and data sources

The Spanish-language version of the PHQ-9 was used to measure depressive symptoms [PHQ-9, 41]. It is a nine-item scale in which each item represents a DSM symptom criterion. Participants are asked to report whether they have experienced the symptom in the last 2 weeks on a Likert scale ranging from 0 to 3, where 0 is “not at all” and 3 is “almost every day,” resulting in a total score ranging from 0 to 27 points [42]. The PHQ-9 was designed to screen for depression and has shown that scores ≥10 have a sensitivity of 88% and specificity of 88% for MDD compared to semi-structured interviews [43]. A diagnostic cutoff of ≥10 is recommended for the detection of MDD; the criteria for classifying severity levels of depression according the PHQ-9 are “moderate” for scores of 14 or below, “moderately severe” for scores ranging between 15 and 19, and “severe depression” for scores of 20 or above. [43].

### Statistical analysis

Descriptive statistics were generated for the total sample. To test for sex differences in total PHQ-9 means, a t-test for independent samples was used.

All possible symptom profiles were identified for PHQ-9 scores equal or higher than 10 points (i.e., clinical sample) using an algorithm of combinatorial optimization. This was calculated using the formula 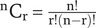 (for formula estimation, see Supplementary Material), which allows calculation of the number of ways of selecting *r* objects out of *n* different objects [44]. The estimation resulted in 382 possible symptom combinations.

#### Theoretical symptom profile analysis

All possible symptom combinations were analyzed for the PHQ-9 using a profiling algorithm developed by Banyard et al. [45]. In this algorithm, individual item responses to the PHQ-9 were dichotomized and coded as either “1” if a symptom was present (a score of 1–3) or “0” if a symptom was absent (a score of 0). Using conditionals, each individual response was matched to their corresponding profile (for details, see Supplementary Material). Different theoretical profiles of depressive symptomatology could thus be constructed yielding a score of 10 or above 382 possible theoretical profiles (for details, see Supplementary Material). Each theoretical profile was assigned a number, and its relative frequency was determined using patient-level data, using a syntax that matches each participant’s PHQ-9 responses to each of the possible 382 theoretical symptom profile combinations. This is a method that prioritizes the identification of *qualitatively distinctive* symptom profiles by emphasizing the absence–presence of symptoms rather than emphasizing *quantitative differences* in their relative scores across each Likert scale. This method was selected to maximize the probability of identifying qualitatively different profiles, since prior research using continuous Likert scale scores to identify latent classes consistently shows that such a method mainly parses cases into quantitatively distinctive subgroups of cases with low-moderate–severe depression [e.g., see 27, 46].

#### Network analysis

Network analysis was used to examine the most prevalent profiles within the relationship between symptoms, so that within a particular network, each *node* represents a PHQ-9 item (i.e., a depression symptom) and each *edge* represents the partial correlation between two symptoms. Network estimation was conducted using pairwise Markov random fields to calculate a nondirected weighted network structure and a Gaussian graphical model to estimate networks with continuous data variables. By using the continuous item scores as inputs into the network model, we were able to comprehensively identify *qualitatively distinctive* profiles (through the prior step of analysis) while examining their *quantitative distinctive* network structures using the full range of Likert scale responses.

The Fruchterman–Reingold algorithm was used to calculate the optimal layout of the networks and to visualize more strongly connected nodes [47]. False-positive relations were excluded by using the “graphical least absolute shrinkage and selection operator” (GLASSO) method, a statistical regularization technique, to increase the specificity of the network [48]. Due to recent developments in network analysis discussing the use of regularized versus non-regularized techniques for the estimation of psychopathology networks [49–51], both types of analysis were conducted, and results are presented in Supplementary Materials. Finally, the extended Bayesian information criterion (EBIC) was used to select the best-fitting model (hyperparameters γ= 0.5 and λ = 0.01).

Strength centrality indexes were calculated for each network. This measure takes the sum of all absolute edge weights to which a node is directly connected [52]. To estimate the network stability, and considering the sample size for each depression symptom profile, a nonparametric bootstrapping procedure was used with 1,000 sample simulations providing results related to the edge-weight accuracy on each network [53, 54]. A case-dropping subset bootstrap was performed for the estimation of the centrality stability, which estimates a correlation stability coefficient (CS coefficient) representing the maximum proportion of the sample that can be dropped and maintaining a 95% probability of a correlation between the original centrality indices and the centrality metric equal or higher to 0.7. Thus, the centrality metric is considered interpretable when the CS coefficient is above 0.25 [53]. All the analyses were performed using “*qgraph”* [55, 56] and “*bootnet”* packages [53, 54] on R studio version 4.0.0 [57].

## Results

### Sample characteristics

The total sample included PHQ-9 data from N = 2,023 participants that had clinically significant depression symptoms (PHQ-9 ≥ 10). Overall, 73.9% (n = 1,495) of participants were female, the mean age of the total sample was 49.87 (*SD* = 17.40) years, and the mean PHQ-9 score was 14.7 (*SD* = 4.36). Approximately 57.7% of participants would be considered moderately depressed (n = 1,168), 26.2% (n = 531) had moderately severe scores, and 16.0% (n = 324) had severe depression. Supplementary Table S1 provides further sample characteristics and details on item-level means and frequencies.

There were no statistically significant differences between female and male participants regarding their mean depression severity scores (t_(2021)_ = −1.51, *p* = .13). In total, 35.5% of participants reported having received a depression diagnosis, and 84.4% of participants who received a diagnosis were women. Approximately 32% (649) reported having previously received or currently were receiving treatment for depression at the time of the assessment. Among participants with a past or current history of treatment, 83.6% (551) were female.

### Symptom profiles

Of the 382 theoretically possible profiles of depressive symptoms operationalized by the profiling algorithm for scores of 10 and above on the PHQ-9, 167 were actually present in the sample. However, more than half of all cases present in the sample (55.6%) were accounted by only eight symptom profiles. Of all 167 profiles present in the sample, the most frequent was profile 1 (n = 510), a “typical” depression profile that includes all nine symptoms of depression measured by the PHQ-9 (all 9 items are positive) and had a frequency of 25.2% of the sample. The mean age for profile 1 was 49.87 (*SD =* 17.40), and the mean PHQ-9 score within this profile was 18.6 (*SD =* 4.85).

The second most frequent profile was profile 2 (n = 205), a profile that includes all symptoms, except for suicidal ideation (item 9), which accounted for 10.1% of cases present in the sample. The mean age for profile 2 was 45.68 (*SD =* 17.41), and the mean PHQ-9 score was 14.88 (*SD =* 4.09).

The third most frequent depressive profile was profile 3 (n = 81), a profile that includes all symptoms except for suicidal ideation (item 9) and psychomotor functioning (item 8, psychomotor retardation or agitation) which accounted for 4% of cases in the sample. The mean age for profile 3 was 44.47 (*SD =* 15.65), and the mean in the PHQ-9 was 13.74 (*SD =* 3.00). Tables 1 and 2 provide further details of the symptom composition and descriptive statistics for the most prevalent profiles. To understand the interaction between symptoms within the three most prevalent profiles, we applied within-profile network analysis.

**Table 1.** Frequency and composition of symptom profiles’ sample (*n* = 2,023)

Symptom profile	Anhedonia	Low mood	Difficulty with sleep	Energy levels	Appetite	Worthlessness	Ability to concentrate	Psychomotor functioning	Suicidal ideation	%	CF%	N
1										25.2	25.2%	510
2										10.1	35.3%	205
3										4.0	39.3%	81
4										3.9	43.2%	79
5										3.6	46.8%	74
6										3.2	50%	65
7										2.9	52.9%	60
8										2.7	55.6%	55

**Table 2.** Descriptive statistics of the eight most frequent theoretical symptom profiles

	Profile 1	Profile 2	Profile 3	Profile 4	Profile 5	Profile 6	Profile 7	Profile 8
	(*N* = 510)	(*N* = 205)	(*N* = 81)	(*N* = 79)	(*N* = 74)	(*N* = 65)	(*N* = 60)	(*N* = 55)
Demographics								
Mean age (*SD*)	49.87 (17.40)	45.68 (17.41)	44.47 (15.65)	46.35 (17.37)	50.36 (17.49)	51.23 (16.18)	48.23 (18.02)	44.60 (15.24)
Female (%)	75%	74%	81%	76%	72%	77%	72%	76%
Mean PHQ–9 (SD)	18.60 (4.85)	14.88 (4.09)	13.74 (3.00)	15.18 (3.96)	13.22 (2.58)	15.37 (3.88)	13.17 (2.64)	13.67 (3.12)
Moderate depression rating	23%	57%	69%	51%	70%	42%	67%	71%
Moderately severe depression rating	34%	26%	25%	32%	27%	40%	33%	20%
Severe depression rating	42%	18%	6%	18%	3%	18%	0%	9%
Diagnostic	40%	40%	27%	43%	30%	43%	35%	24%
Treatment	37%	32%	31%	44%	34%	42%	22%	27%

*Note*: “Diagnostic” is used to identify participants who self-reported having received a diagnosis of depression. On the other hand, “treatment” is assigned to participants currently undergoing depression treatment.

### Network analysis

#### Profile 1: Typical depression

This profile comprises participants that present all of the typical symptoms of depression, which means the presence of anhedonia, low mood, sleep problems, low energy, appetite changes, worthlessness, concentration problems, psychomotor functioning (psychomotor retardation or agitation), and suicidal ideation.

**Figure 1.**
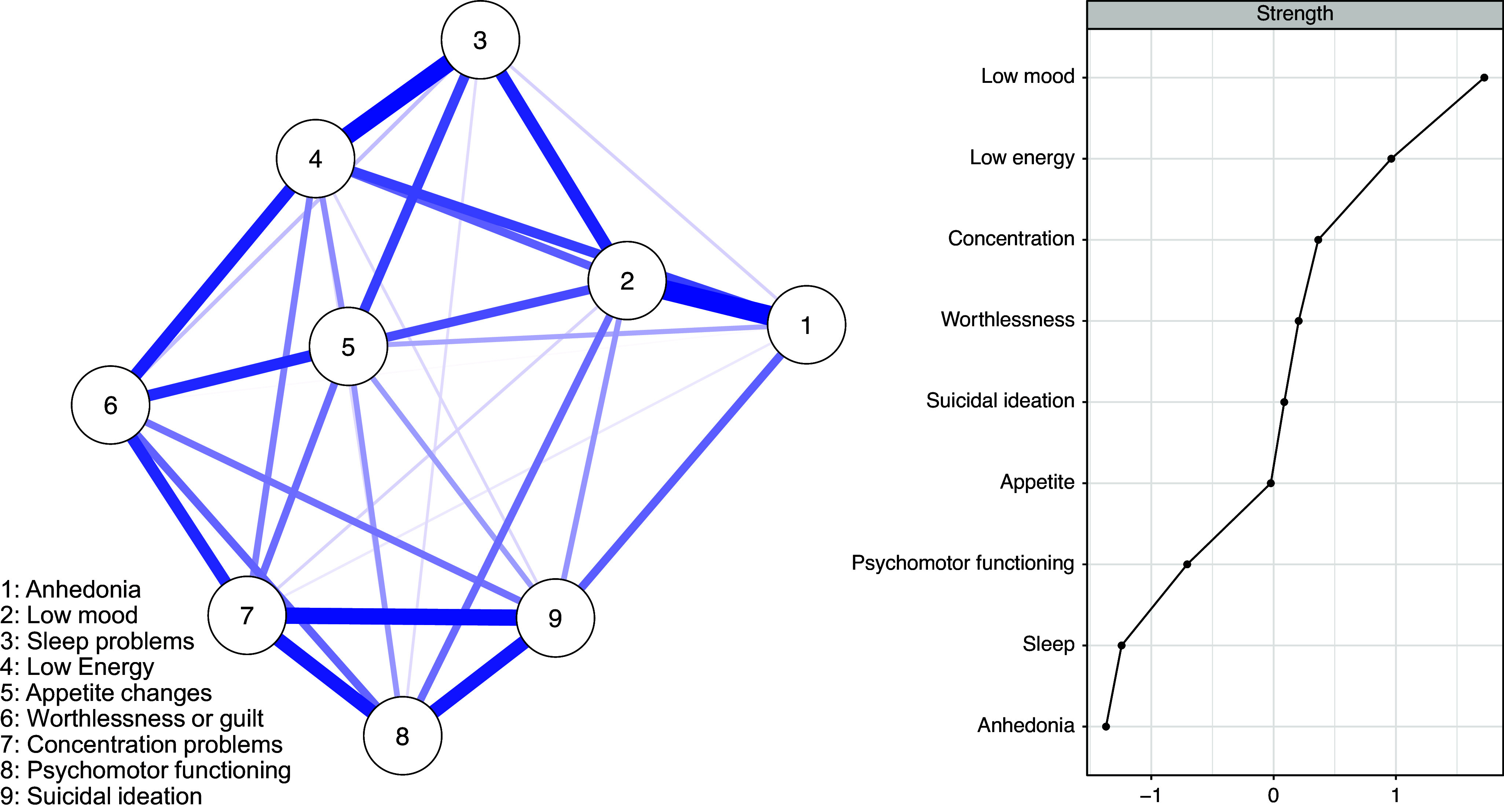
Network of symptoms and centrality plot for profile 1 with all of the depressive symptoms. *Note*: The centrality plot shows standardized strength indices.

The network of profile 1 is visualized in Figure 1 and shows a strong positive connection between low mood and anhedonia (*pr* = 0.30) and also between sleep problems and low energy (*pr* = 0.26). Also, there is a community of tightly interrelated symptoms including suicidal ideation–concentration problems (*pr* = 0.24), suicidal ideation–changes in psychomotor functioning (agitation or retardation) (*pr* = 0.20), and concentration problems–changes in psychomotor functioning (*pr* = 0.20).

The nodes with the highest strength centrality in profile 1 were low mood, low energy, and concentration problems. The least central nodes in terms of strength centrality were anhedonia and sleep problems. Node strength centrality demonstrated an interpretable level of stability (*CS* (cor = 0.7) = 0.36). Details of the centrality stability test are shown in Supplementary Figures S1 and S2.

#### Profile 2: Typical depression without suicidal ideation

This profile included all typical depression symptoms except for suicidal ideation.

**Figure 2.**
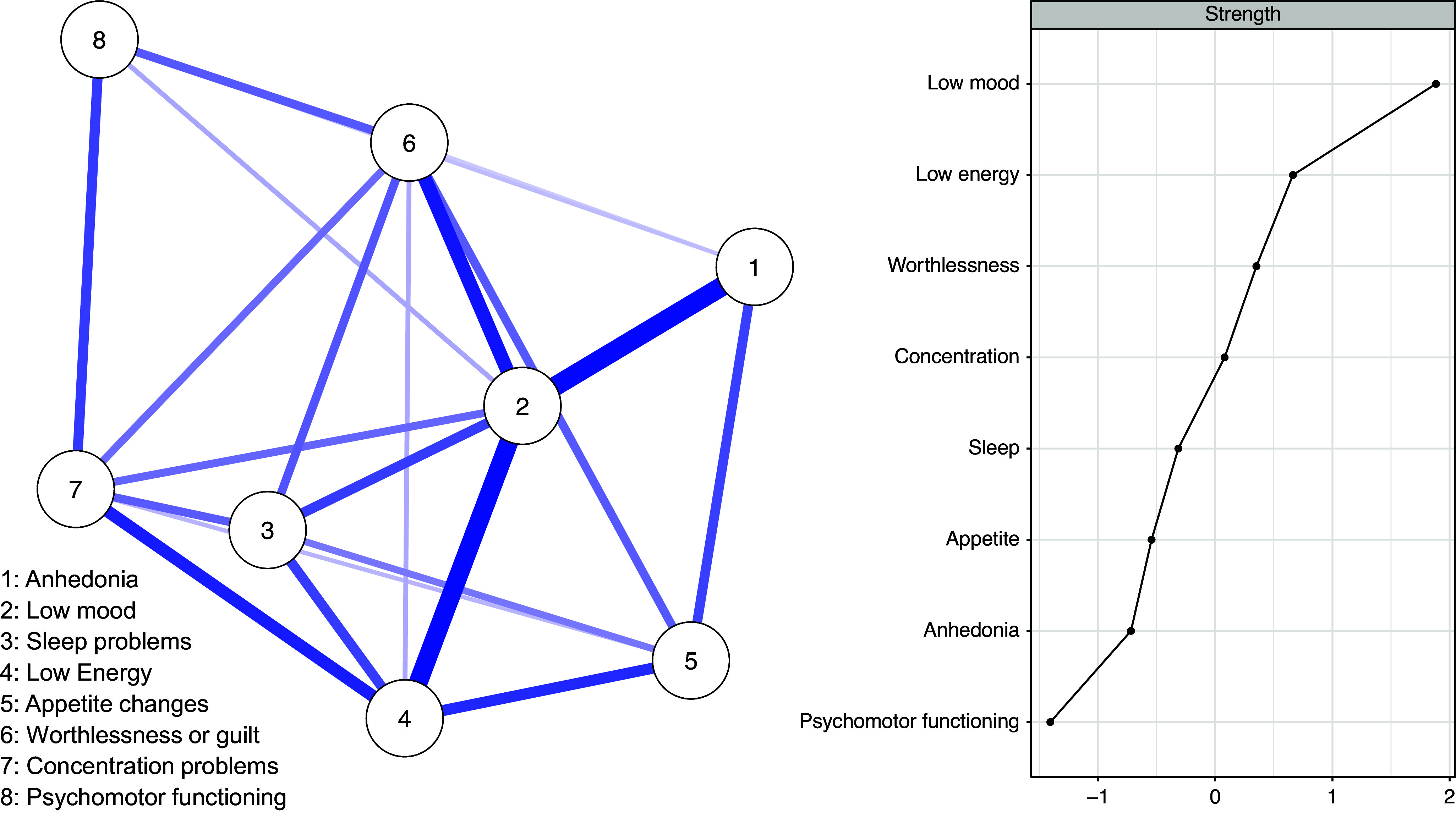
Network of symptoms and centrality plot for profile 2. *Note*: The centrality plot shows standardized strength indices.

The network of profile 2 is visualized in Figure 2 and shows a strong positive connection between low mood and anhedonia (*pr* = 0.27), low mood and low energy (*pr* = 0.26), and low mood and worthlessness (*pr* = 0.18). The nodes with the highest strength centrality were low mood, low energy, and worthlessness. The least central nodes in terms of strength centrality were psychomotor functioning and anhedonia. These data must be interpreted with caution because node strength centrality demonstrated low stability (*CS* (cor = 0.7) = 0.12). See details of the centrality stability test in Supplementary Figures S4 and S5.

#### Profile 3: All depressive symptoms except for psychomotor functioning and suicidal ideation

This profile includes all PHQ-9 symptoms except for suicidal ideation and changes related to psychomotor functioning.

**Figure 3.**
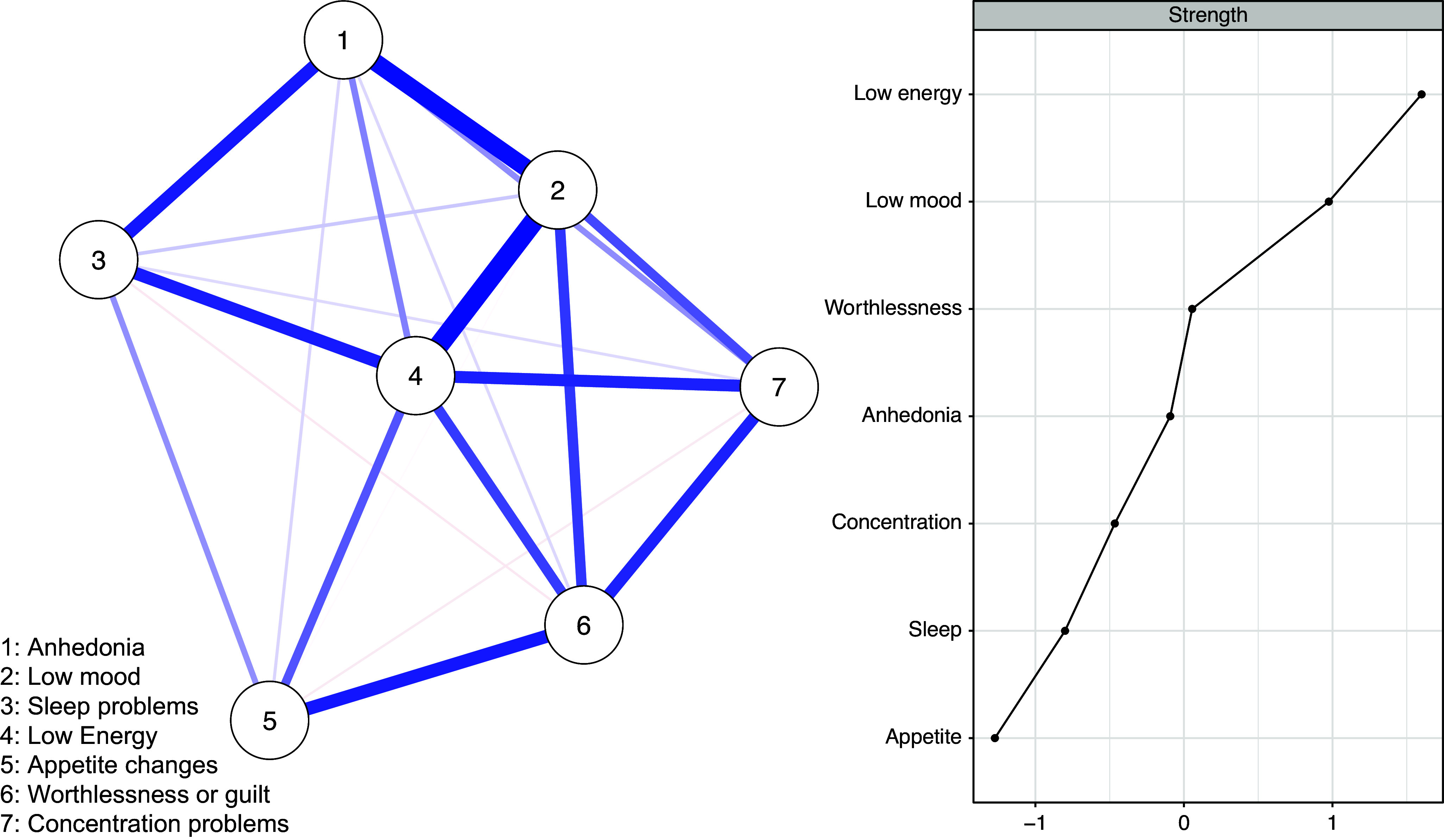
Network of symptoms and centrality plot for profile 3. *Note*: The centrality plot shows standardized strength indices.

The network of profile 3 is visualized in Figure 3. Profile 3 was the third most frequent in the sample. Due to the small sample size (n = 81), this network was estimated using a threshold of null 0.05 instead of applying the GLASSO method (which did not converge in this subgroup). The network shows a strong positive connection between low mood and low energy (*pr* = 0.37), low mood and anhedonia (*pr* = 0.32), sleep problems and anhedonia (*pr* = 0.23), sleep problems and low energy (*pr* = 0.22), and appetite and worthlessness (*pr* = 0.23).

The nodes with the highest strength centrality were low energy, low mood, and worthlessness. In contrast, the nodes with the least strength centrality were sleep problems and appetite changes. However, these data must be interpreted with caution because node strength centrality demonstrated low stability related to the sample size (*CS* (cor = 0.7) = 0.21; for details on the centrality stability test and accuracy, see Supplementary Figures S7 and S8).

**Figure 4.**
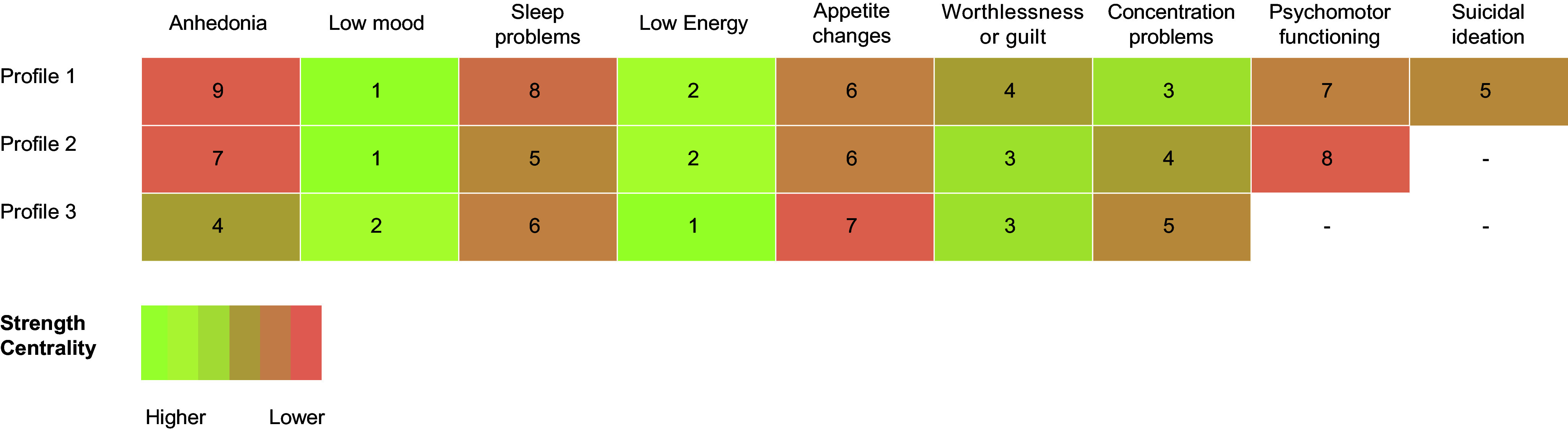
Strength centrality rankings’ indices for the three most prevalent profiles. *Note*: Numbers indicate strength centrality rankings. Profile 1: all of the typical symptoms of depression; profile 2: typical depression without suicidal ideation; profile 3: all depressive symptoms except for psychomotor functioning and suicidal ideation. Figure adapted with permission from Malgaroli et al. [40] and license provided by Elsevier.

Results indicated that node centrality varied across the most frequent profiles of depression (see Figure 4 for a comparison). Consistently, the most central symptoms were low mood and low energy, and the less central symptoms were anhedonia, change in the psychomotor functioning, and appetite changes.

## Conclusions

The present study identified different depressive symptom profiles and examined their network structure using PHQ-9 data from participants with clinically relevant depressive symptoms drawn from three community samples. Results show that 167 of the 382 theoretically possible symptom combinations were present in the sample, and 55.6% of all profiles were accounted for by only eight profiles. The most frequent symptom profile included all typical symptoms of depression measured by the PHQ-9 (25.2%). These results are consistent with studies that applied a similar approach using patient-level data, which show that many cases display similar symptom profiles. For example, Zimmerman et al. [13] similarly found in a community sample that out of the 227 symptom combinations calculated using semi-structured interviews (Structured Clinical Interview for DSM-IV, SCID-I), just 170 were empirically observed and concluded that nine combination profiles accounted for the depression symptoms of 40% of patients. These findings align with those of Park et al. [39], who identified, in a clinical sample, 119 symptom combinations within their sample. Both studies, using a different approach from the one used in the present study, concluded that combinatorial patterns with all nine symptoms of depression were the most prevalent in samples from the USA and South Korea [13, 39]. Overall, the extent of diagnostic heterogeneity observed empirically within clinical and community-based samples is lower than that has been previously suggested based on theoretical arguments [e.g., 16].

The three most prevalent profiles showed similar mean levels of overall symptom severity on the PHQ-9 total score. However, there were evident differences in their centrality indices and in the interrelations between symptoms. This is clinically relevant, considering that one of these profiles shows suicidal ideation and is rated with the same severity as the other profiles, supporting the idea that looking at total scores in scales omits important qualitative differences between symptoms concerning their hierarchy and clinical relevance [28, 58].

Regarding the relationship between symptoms, there are several differences between the profiles related to the centrality indices and the connection between them. The most common profile, namely profile 1, showed a strong relationship between concentration problems, suicidal ideation, and psychomotor functioning, with concentration problems constituting a rather strong node within this profile. This is different to those profiles that do not include suicidal ideation. These results are in line with those reported in two meta-analyses that found an association between the attentional process and suicidal spectrum behaviors [59, 60]. Thus, this profile could be relevant in identify vulnerable people in the population because it has been highlighted that sad mood and concentration problems, the two most central symptoms for this profile, are the most disabling symptoms of depression [34].

Another difference between the profiles is present in profile 2, which shows a strong connection between low mood, low energy, and self-perception. In this profile, with all of the symptoms except for suicidal ideation, worthlessness takes a key role in comparison to profile 1 which includes all of the symptoms. On the other hand, in profile 3, worthlessness is strongly related to changes in appetite, which is unique to this profile. Profile 3 is characterized by the centrality of low energy which is different from profiles 1 and 2, where low mood is the most central symptom.

In the most frequent profiles of depressive symptoms, results show that low mood and low energy are consistently among the three most central symptoms; this is similar to the results reported in a systematic review that considered the results of 58 cross-sectional depression networks. Interestingly, anhedonia does not appear as a central node on these profile networks, even though it has a strong positive connection with low mood (*pr* = 0.30, 0.27, 0.21), showing a consistent relationship on the three profiles analyzed. This is also consistent with previous studies that found the connection between low mood and anhedonia was the networks’ most frequent and robust edge [40]. This is theoretically interesting, considering that anhedonia has been conceived as a main symptom according to the DSM diagnostic criteria for MDD.

In terms of methodology, there are limitations related to the sample sizes for each profile subsample that must be considered when interpreting these results. The estimation method could impact the visualization of the networks for small sample sizes, generating networks that overfit to the data and impacting the stability of the centrality indexes [53]. Another limitation of this study is the use of cross-sectional data, which provide only a static vision of the profile symptoms that could change over time. Future studies should consider these limitations and explore the relationship between symptoms over time using longitudinal designs with repeated measures. Also, it could be relevant to understand the possible directional influence between the symptoms considering time series data. An additional limitation of this study, as well as depression heterogeneity research, that has been shown in previous studies [61] is that different instruments can assess different symptoms of depression. Therefore, this study captures the heterogeneity of the specific screening instrument that was used, and other instruments that capture additional symptoms or phrase the same symptoms differently may yield different heterogeneity profiles. Consequently, no claims can be made about substantive heterogeneity as it occurs in nature (i.e., carving nature at its joints) but rather as it emerges from the use of the PHQ-9, a widely used screening measure and recommended as a preferred measure for the screening of depression [62, 63]. It is important to acknowledge that finding common ground for the screening of depression by utilizing one instrument also may have a negative impact on the efforts to map out heterogeneity; it is easier to aggregate findings from different studies but all researchers are looking through the same lens that could narrow the comprehension of depression [64, 65].

Even with these limitations, this study is the first to our knowledge that combined the identification of qualitatively distinctive symptom profiles and examined the network structure of such profiles using network analyses. Previous network analysis research has shown the relevance of investigating the interrelations between symptoms of depression [40]. While the present results support this approach, they also expand previous research about network analysis and depression and provide empirical support regarding the relevance of looking at the different profiles and the different relations between symptoms for each one. Also, these results could be relevant considering treatment personalization.

## Supporting information

Núñez et al. supplementary material Núñez et al. supplementary material

## Supplementary material

The supplementary material for this article can be found at https://doi.org/10.1192/j.eurpsy.2024.1756.
