# Supplementary material for: Understanding symptom profiles of depression with the PHQ-9 in a community sample using network analysis
Source: Eur Psychiatry. 2024 May 23;67(1):e50. doi: 10.1192/j.eurpsy.2024.1756 (PMC11441345; doi:10.1192/j.eurpsy.2024.1756)
Supplement: Núñez et al. supplementary material [file S0924933824017565sup001.pdf]

Supplementary material

Understanding Symptom Profiles of Depression with the PHQ-9 in a Community Sample Using Network Analysis

Table 1

Descriptive statistics (N=2,023)

	Total sample (n=2,023)				Women (n= 1,495)			Men (n= 528)		
Item	M	SD	median	mode	M	SD	median	M	SD	median
Anhedonia	1.76	0.99	2	1	1.79	0.97	2	1.69	1.03	2
Low mood	1.86	0.93	2	1	1.87	0.92	2	1.83	0.96	2
Difficulty with sleep	2.12	0.99	2	3	2.15	0.99	3	2.03	0.98	2
Energy levels	2.21	0.88	2	3	2.25	0.87	3	2.09	0.90	2
Appetite	1.77	1.08	2	3	1.79	1.08	2	1.72	1.09	2
Worthlessness	1.48	1.13	1	1	1.49	1.13	1	1.48	1.13	1
Ability to concentrate	1.39	1.12	1	1	1.39	1.13	1	1.40	1.12	1
Speed of functioning	1.29	1.12	1	0	1.26	1.12	1	1.36	1.12	1
Suicidal ideation	0.90	1.03	1	0	0.88	1.02	1	0.94	1.07	1
PHQ-9 total	14.78	4.36	14	10	14.32	4.45	14	14.54	4.35	13
Age	49.87	17.40	51	54	50.41	16.81	51	48.21	18.90	49

Table 2
Descriptive statistics profile 1 (all symptoms of depression)

	Total sample profile 1 (n=510)		
Item	M	SD	median
Anhedonia	2.00	0.83	2
Low mood	2.07	0.83	2
Difficulty with sleep	2.25	0.79	2
Energy levels	2.25	0.80	2
Appetite	2.11	0.83	2
Worthlessness	2.10	0.82	2
Ability to concentrate	2.02	0.82	2
Speed of functioning	1.97	0.84	2
Suicidal ideation	1.83	0.84	2
PHQ-9 total	18.6	4.85	18
Age	49.87	17.40	51

Figure 1

Accuracy and stability of edge-estimates profile 1 (1000 simulation, non-parametric bootstrap, grey area represent boostraped CIs)

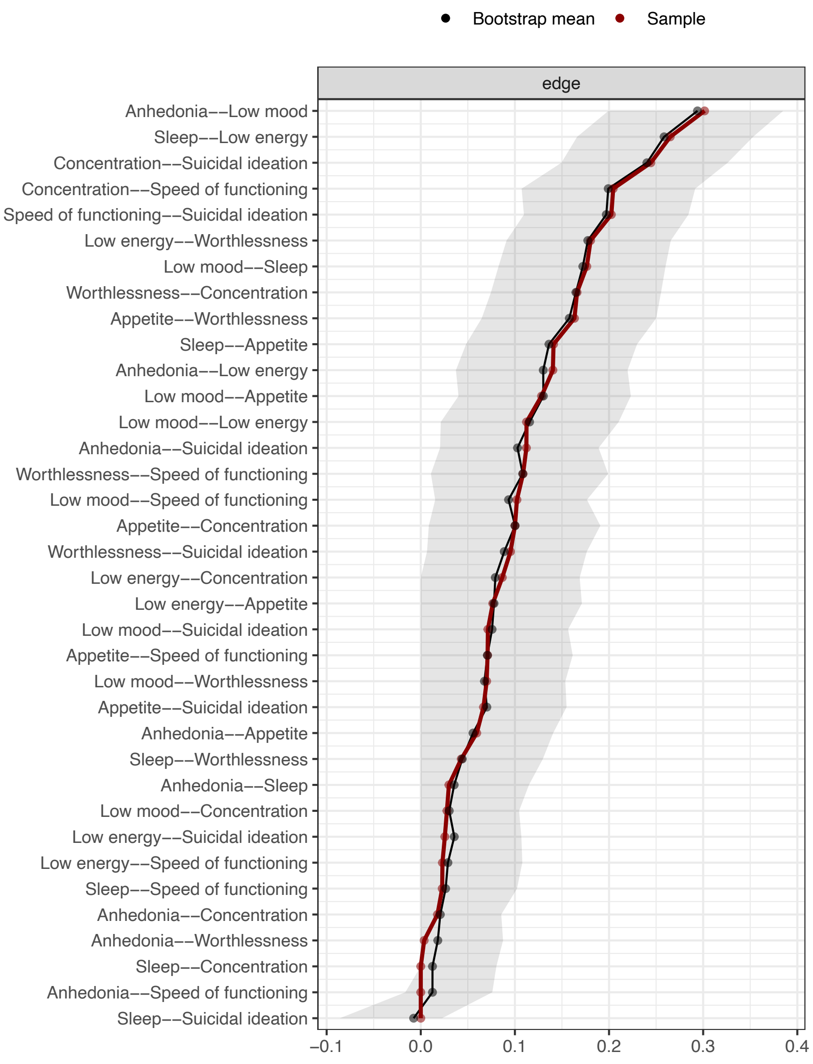

Figure 2

Plot Centrality stability test for the profile 1 (N= 510).

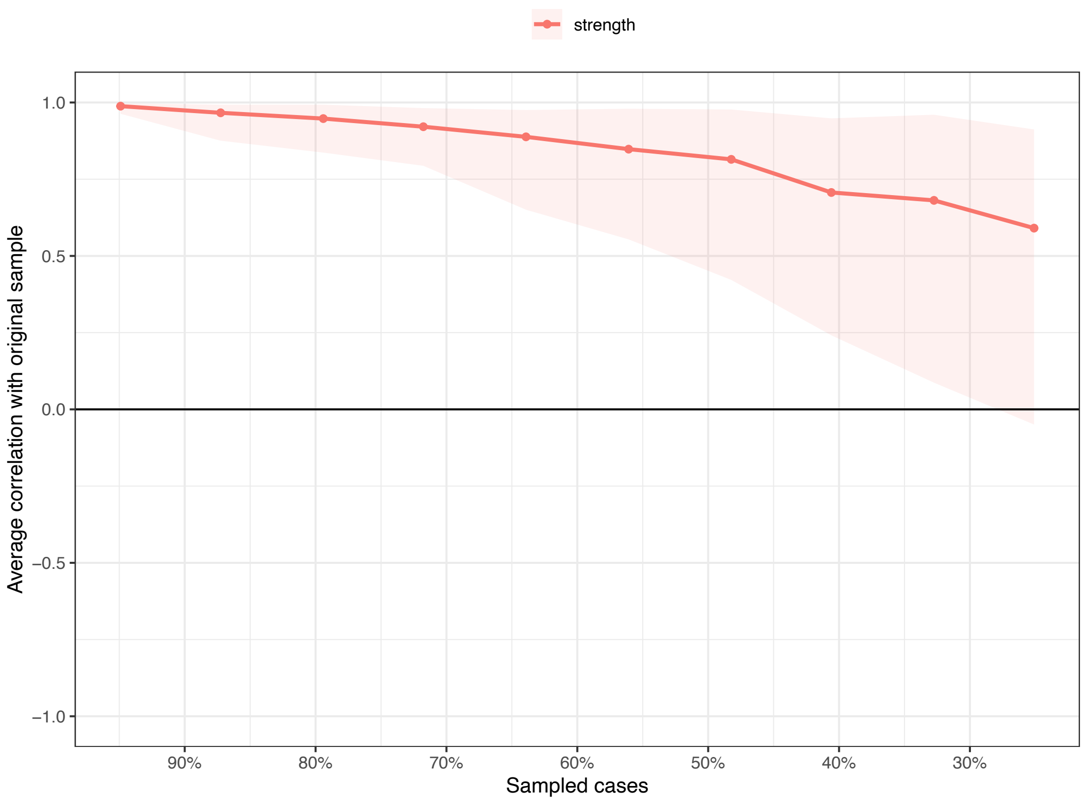

Figure 3
Bootstrapped difference test profile 1

A. Significant differences in edge-weights

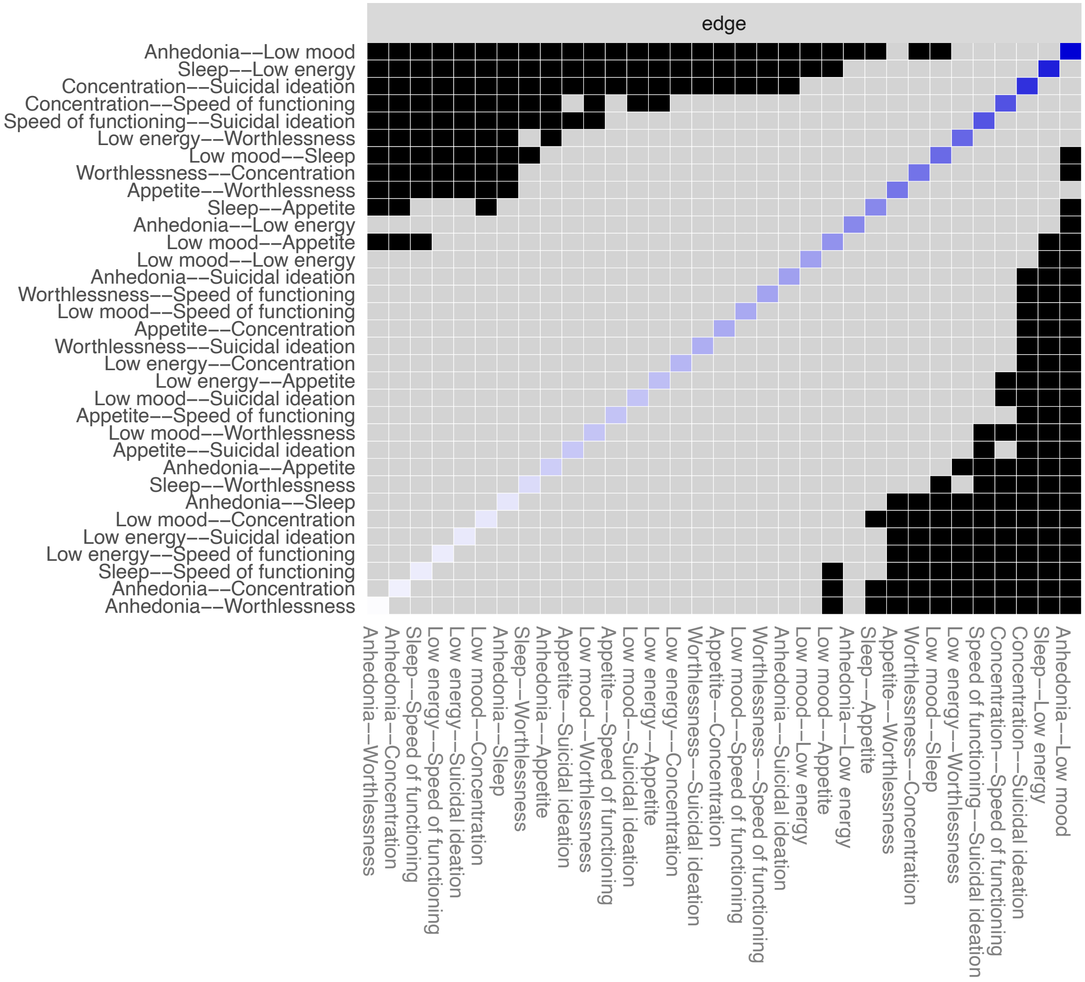

B. Significant differences in node strength

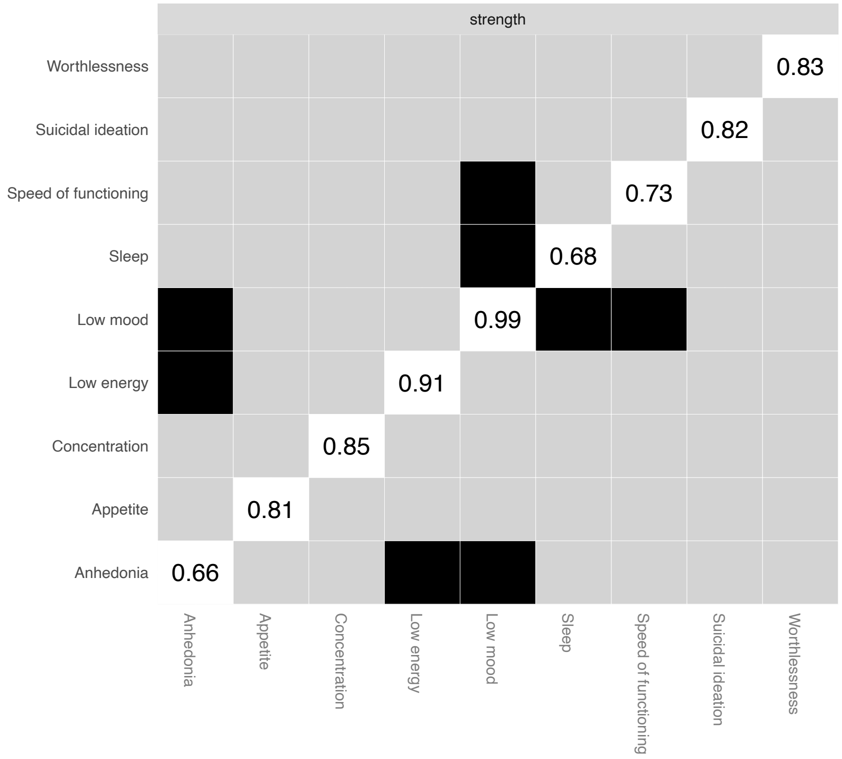

Table 3
Descriptive statistics profile 2

	Total sample profile 2 (n= 205)		
Item	M	SD	median
Anhedonia	1.78	0.86	1
Low mood	1.76	0.83	2
Difficulty with sleep	2.15	0.86	2
Energy levels	2.07	0.85	2
Appetite	1.88	0.90	2
Worthlessness	1.81	0.87	2
Ability to concentrate	1.73	0.86	1
Speed of functioning	1.72	0.84	1
PHQ-9 total	14.88	4.09	14
Age	45.68	17.41	46

Figure 4

Accuracy and stability of edge-estimates profile 2 (1000 simulation, non-parametric bootstrap)

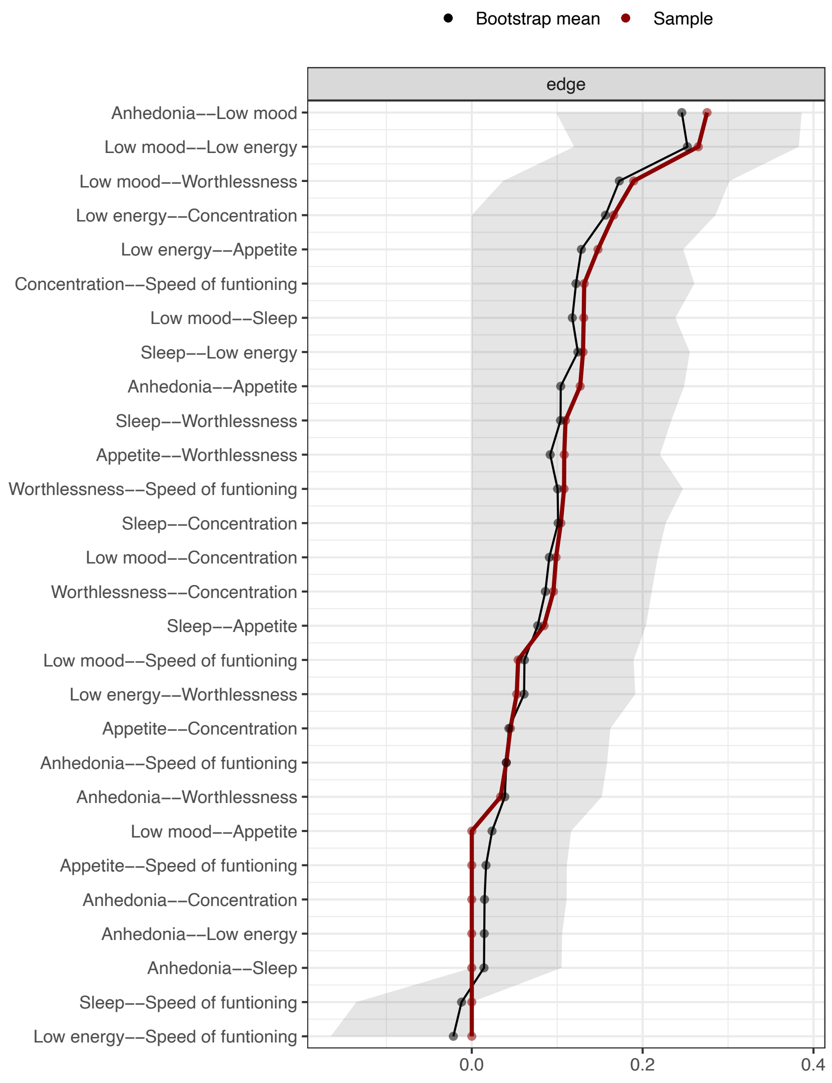

Figure 5
Centrality stability test for the profile 2 (N= 205)

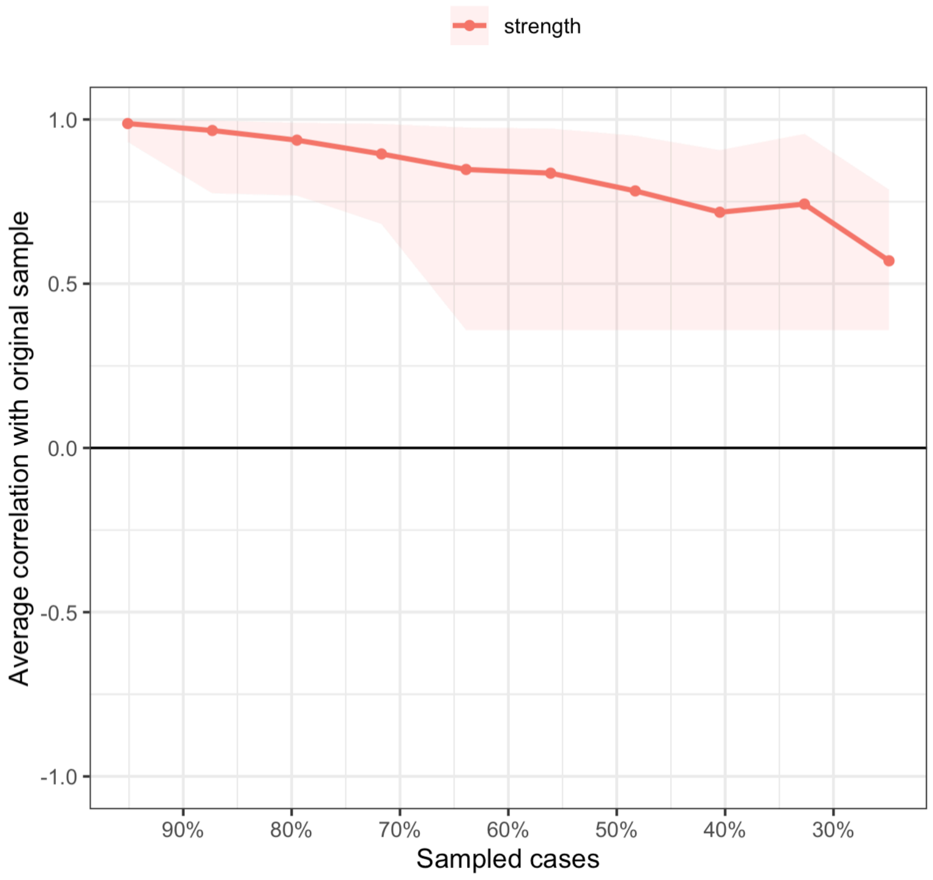

Figure 6

Edge-weights difference test profile 2.

A. Significant differences in edge-weights

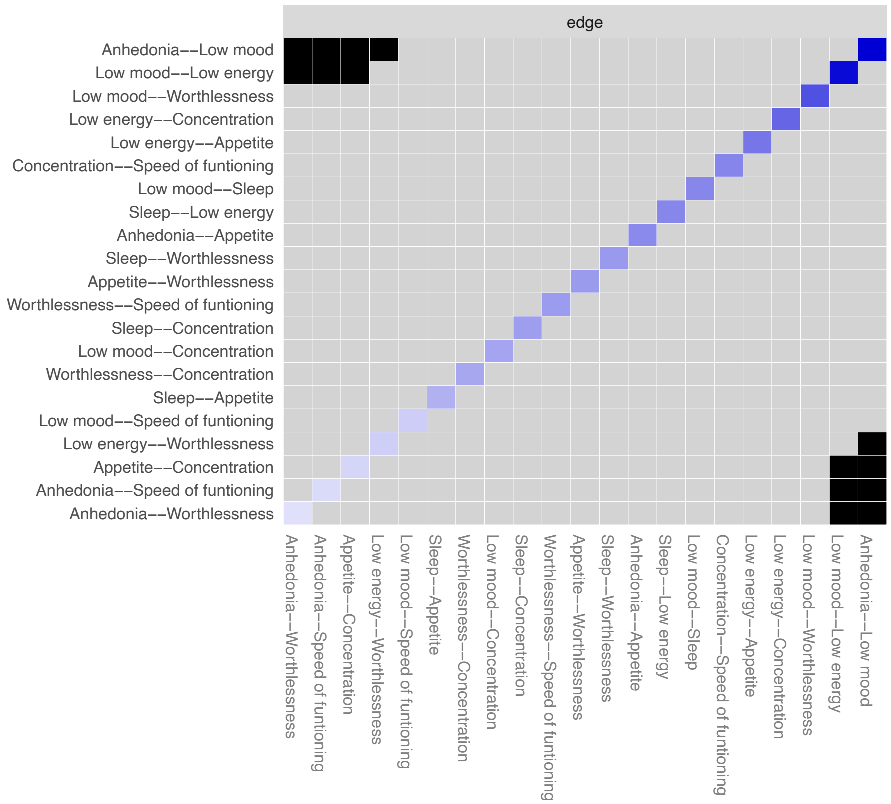

B. Significant differences in node strength

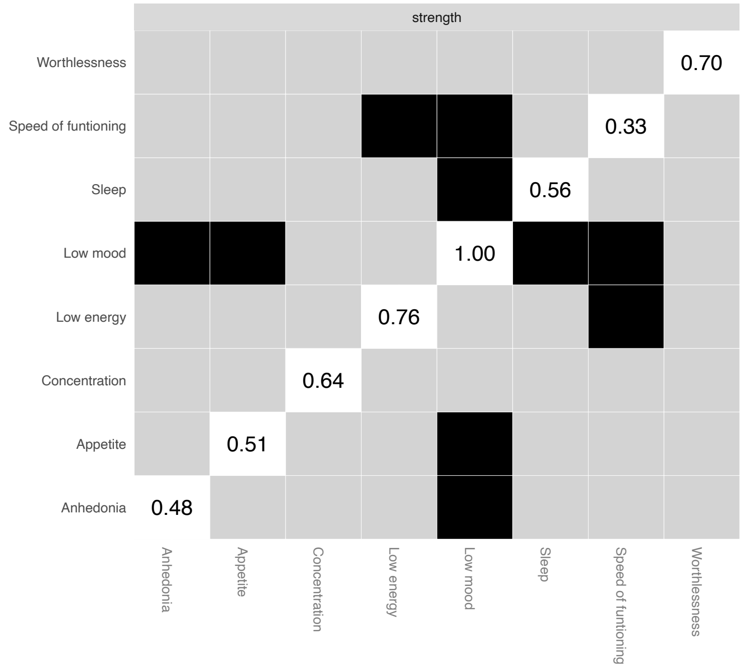

Table 6
Descriptive statistics profile 3

	Total sample profile 3 (n= 81)		
Item	M	SD	median
Anhedonia	1.88	0.87	2
Low mood	1.73	0.87	1
Difficulty with sleep	2.31	0.85	3
Energy levels	2.28	0.81	3
Appetite	2.06	0.81	2
Worthlessness	1.73	0.77	2
Ability to concentrate	1.75	0.87	1
PHQ-9 total	13.74	3.00	13
Age	44.47	15.65	44

Figure 7

Accuracy and stability of edge-estimates profile 3 (1000 simulation, non-parametric bootstrap)

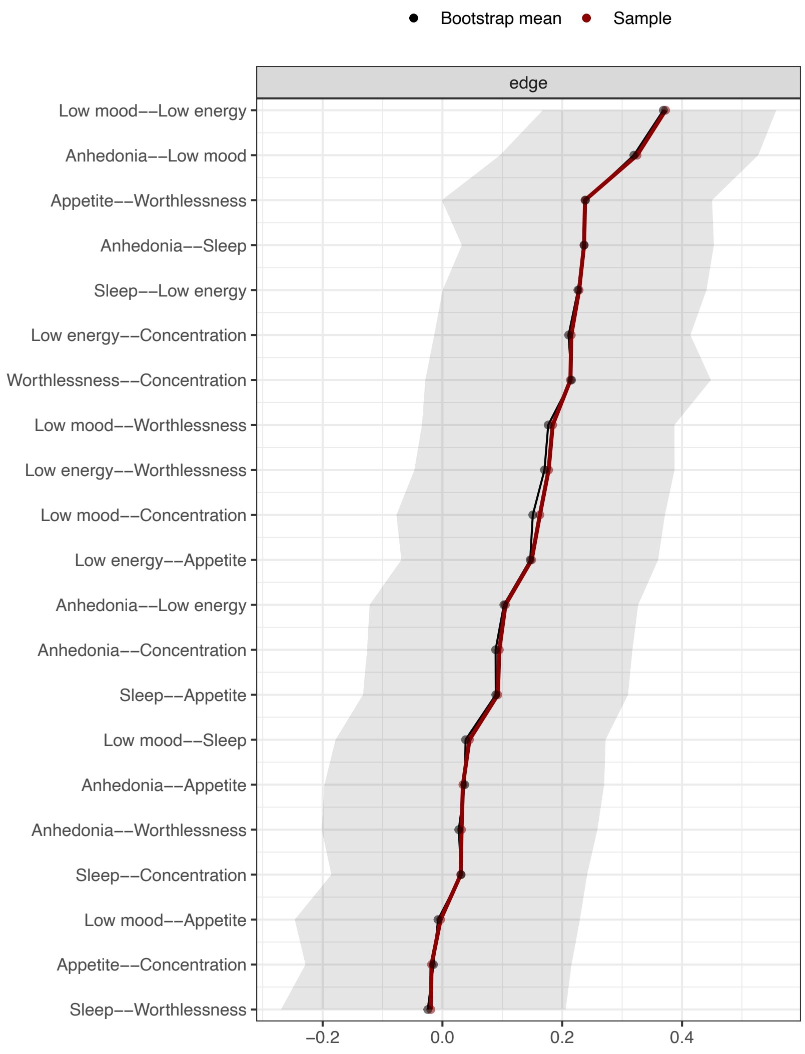

Figure 8
Centrality stability test for the profile 3 (N=81).

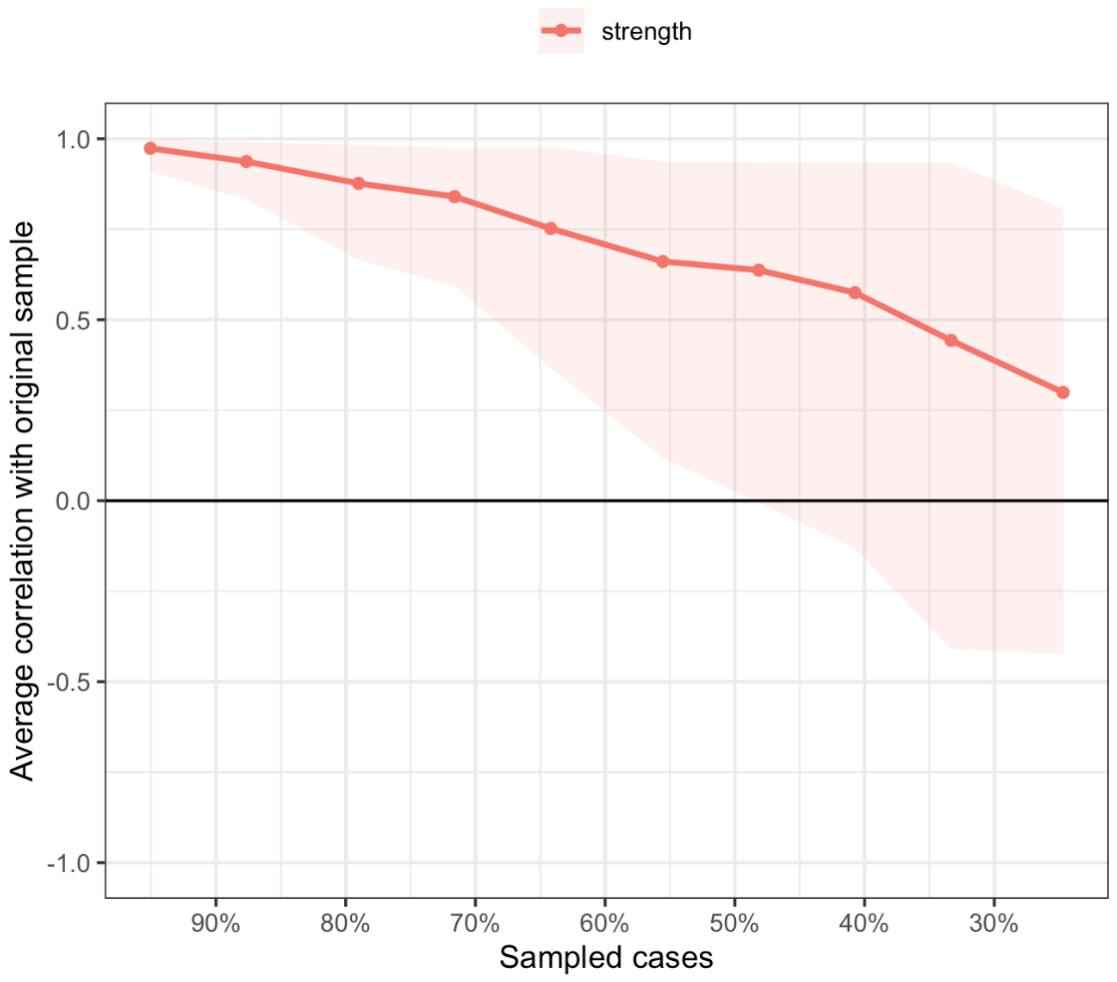

Figure 9

Edge-weights difference test profile 3.

A. Significant differences in edge-weights

B. Significant differences in node strength

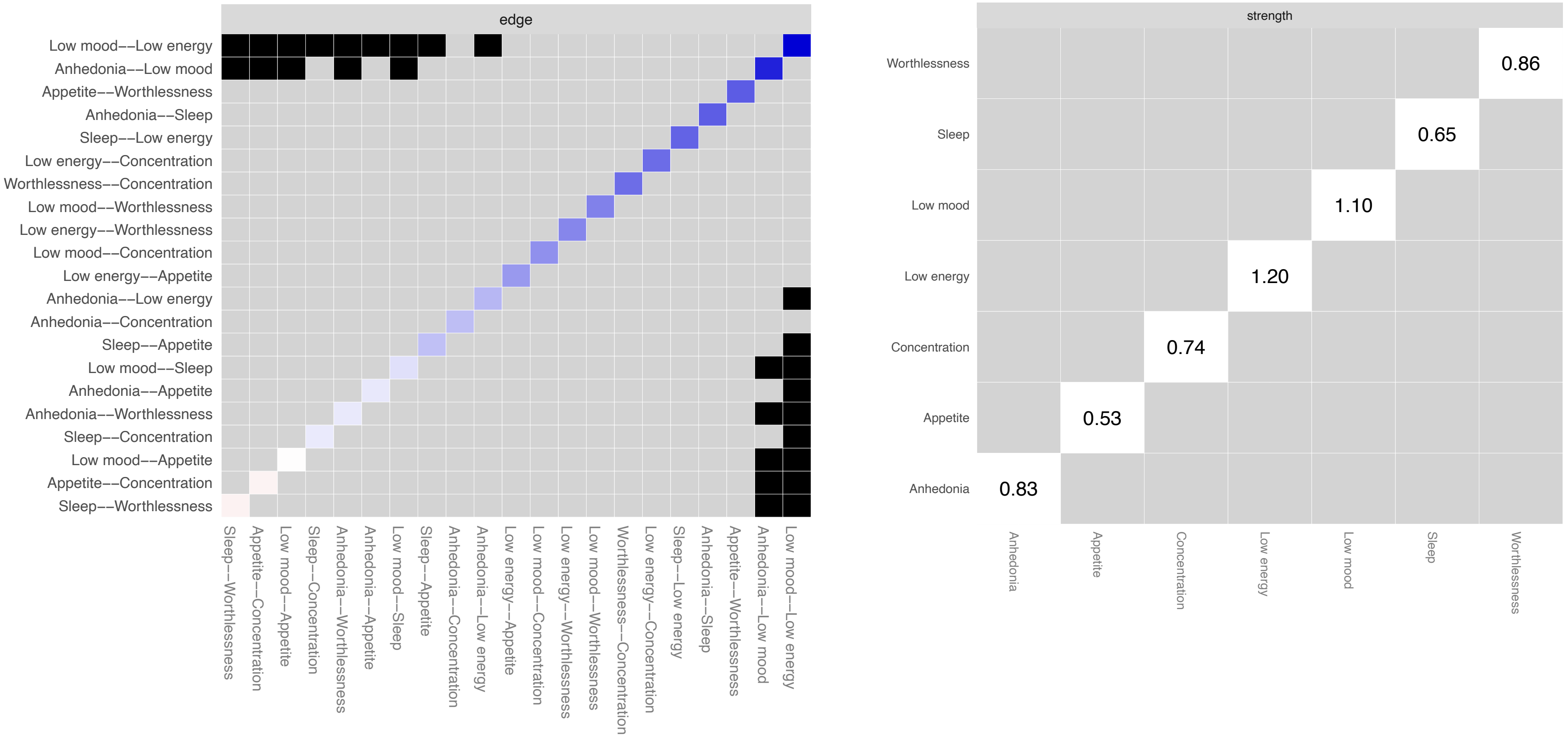

Table 6

Estimation of total number of combination of symptoms using the PHQ-9

An algorithm combinatorial optimization, nCr formula, was used to calculated all the possible symtoms (idems) combination using the PHQ-9 with a 10 point cut-off threshold. This formula calculated number of possible combinations when r objects are selected out of n different objects is given. 511 symptoms combinations are possible using the total score of the PHQ-9, when we consider just scores over 10 point, this variability is reduced to 382 profiles.

Symptom used in combinatorial formula	nCr formula<br>nCr = n! / r! (n - r)!
4 symptoms	9C4 = 9! / 4! (9-4)! = 126
5 symptoms	9C5 = 9! / 5! (9-5)! = 126
6 symptoms	9C6 = 9! / 6! (9-6)! = 84
7 symptoms	9C6 = 9! / 7! (9-7) = 36
8 symptoms	9C8 = 9! / 8! (9-8) = 9
9 symptoms	9C9 = 9! / 9! (9-9) = 1
Total symptoms combinations for cutoff point over 10 on PHQ-9	382

Note: nCr formula for the number of possible combinations when r objects are selected out of n different objects is given (for further information about the use of nCr formula see *Nath Banerjee, S. (2020). THE FORMULA nCr REVISITED. Journal of Mathematical Sciences & Computational Mathematics, 1(1), 75-78.*)

Figure 10
Profiles algorithm R code example

```
PHQ9_i1==1 & PHQ9_i2==0 & PHQ9_i3==0 & PHQ9_i4==0 & PHQ9_i5==0 & PHQ9_i6==0 &PHQ9_i7==0 &
PHQ9_i8==0 & PHQ9_i9==0 ~ "1"
```

The original algorithm, developed by Banyard et al. (upcoming publication), was initially implemented on SPSS. For the analysis conducted, the original Likert scale response options ranging from 0 to 3 were transformed into a binary variable (1 = endorsed, 0 = not endorsed), where endorsement indicates the presence of a depressive symptom.

This algorithm, which encompasses all 511 possible symptoms and their corresponding responses on the PHQ-9, categorizes each participant's responses into a combination profile using conditional statements. For this study the original algorithm was adapted to be used on R. This was achieved by employing conditionals AND and OR to classify each participant's individual responses into all possible combinations.

Table 7

Comparison non-regularized and regularized network profile 1

Non-regularized network profile 1<br>(GGMnonreg)<br>layout = circle	Regularized network profile 1<br>GLASSO (qgraph)<br>layout = circle
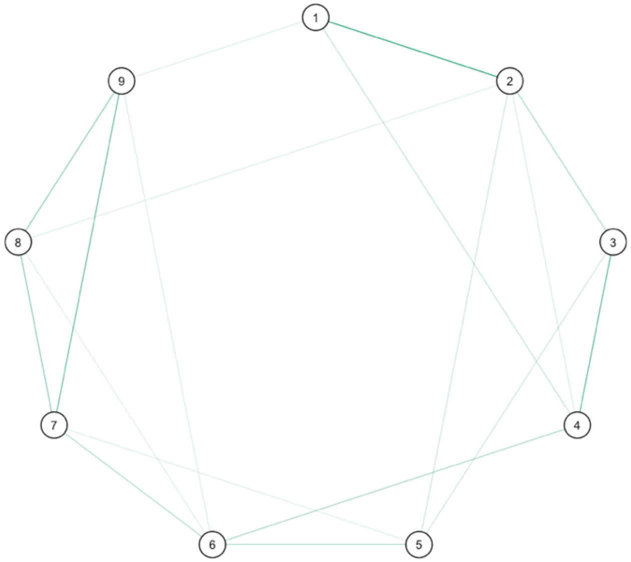	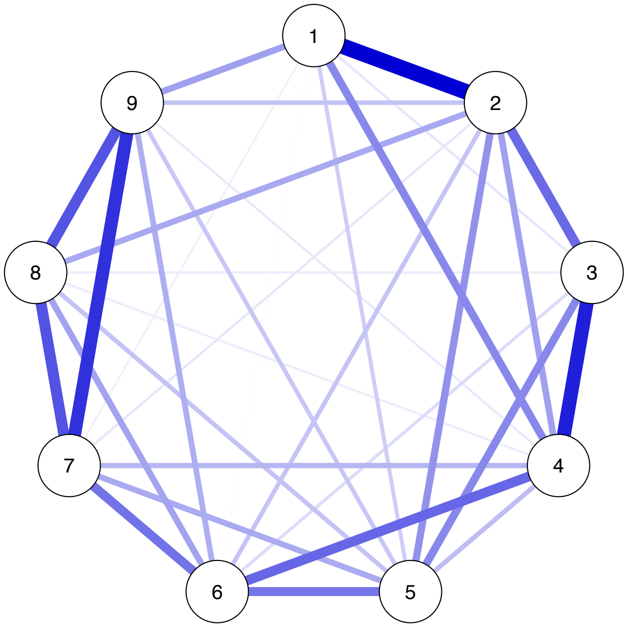

Table 8

Comparison Adjacency matrix non-regularized and regularized network profile 1

Non-regularized GGM network (GGMnonreg)

	phq9_1	phq9_2	phq9_3	phq9_4	phq9_5	phq9_6	phq9_7	phq9_8	phq9_9
phq9_1	0.0000000	0.3079941	0.0000000	0.1454407	0.0000000	0.0000000	0.0000000	0.0000000	0.1136092
phq9_2	0.3079941	0.0000000	0.1767102	0.1156402	0.1344702	0.0000000	0.0000000	0.1044993	0.0000000
phq9_3	0.0000000	0.1767102	0.0000000	0.2556306	0.1324487	0.0000000	0.0000000	0.0000000	0.0000000
phq9_4	0.1454407	0.1156402	0.2556306	0.0000000	0.0000000	0.1794010	0.0000000	0.0000000	0.0000000
phq9_5	0.0000000	0.1344702	0.1324487	0.0000000	0.0000000	0.1643139	0.1018292	0.0000000	0.0000000
phq9_6	0.0000000	0.0000000	0.0000000	0.1794010	0.1643139	0.0000000	0.1685972	0.1029723	0.1070210
phq9_7	0.0000000	0.0000000	0.0000000	0.0000000	0.1018292	0.1685972	0.0000000	0.2083996	0.2457092
phq9_8	0.0000000	0.1044993	0.0000000	0.0000000	0.0000000	0.1029723	0.2083996	0.0000000	0.2078215
phq9_9	0.1136092	0.0000000	0.0000000	0.0000000	0.0000000	0.1070210	0.2457092	0.2078215	0.0000000

Regularized GGM network (glasso; qgraph)

	phq9_1	phq9_2	phq9_3	phq9_4	phq9_5	phq9_6	phq9_7	phq9_8	phq9_9
phq9_1	0.00000000	0.3014894	0.0298990	0.1403398	0.0594649	0.00359186	0.0177367	0.0000000	0.1122188
phq9_2	0.30148944	0.0000000	0.1763758	0.1123558	0.1283158	0.06999727	0.0277888	0.1021575	0.0713098
phq9_3	0.02989905	0.1763758	0.0000000	0.2650284	0.1410580	0.04301459	0.0000000	0.0227379	0.0000000
phq9_4	0.14033985	0.1123558	0.2650284	0.0000000	0.0765241	0.18039721	0.0865984	0.0230282	0.0255691
phq9_5	0.05946495	0.1283158	0.1410580	0.0765241	0.0000000	0.16326254	0.1000848	0.0709834	0.0666288
phq9_6	0.00359186	0.0699972	0.0430145	0.1803972	0.1632625	0.00000000	0.1660193	0.1086909	0.0953083
phq9_7	0.01773677	0.0277888	0.0000000	0.0865984	0.1000848	0.16601933	0.0000000	0.2044401	0.2444062
phq9_8	0.00000000	0.1021575	0.0227379	0.0230282	0.0709834	0.10869094	0.2044401	0.0000000	0.2024414
phq9_9	0.11221881	0.0713098	0.0000000	0.0255691	0.0666288	0.09530831	0.2444062	0.2024414	0.0000000

Table 9

Comparison non-regularized and regularized network profile 2

Non-regularized network profile 2<br>(GGMnonreg)<br>layout = circle	Regularized network profile 2<br>GLASSO (qgraph)<br>layout = circle
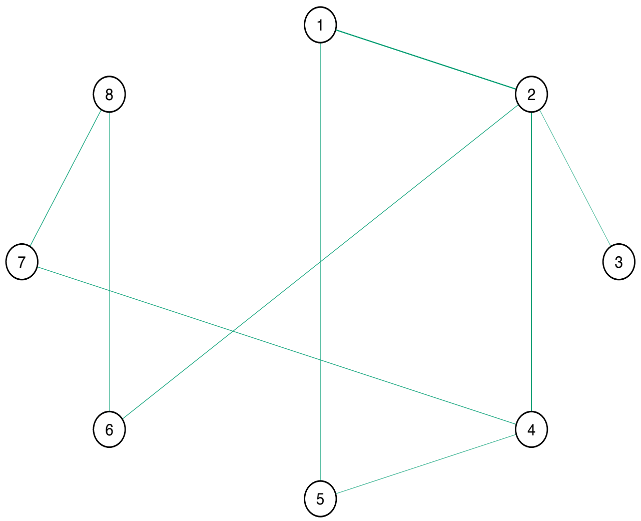	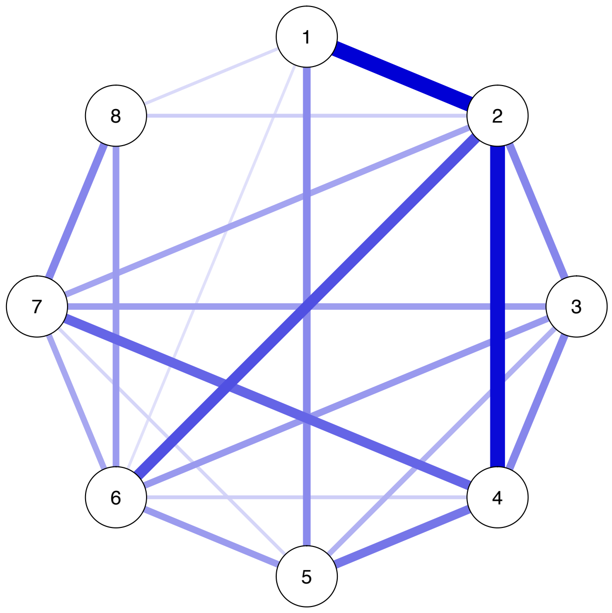

Table 10

Comparison Adjacency matrix Non-regularized and regularized networks GLASSO profile 2

Non-regularized GGM network (GGMnonreg)

	phq9_1	phq9_2	phq9_3	phq9_4	phq9_5	phq9_6	phq9_7	phq9_8
phq9_1	0.0000000	0.3152263	0.0000000	0.0000000	0.1596752	0.0000000	0.0000000	0.0000000
phq9_2	0.3152263	0.0000000	0.1645508	0.3089800	0.0000000	0.2057773	0.0000000	0.0000000
phq9_3	0.0000000	0.1645508	0.0000000	0.0000000	0.0000000	0.0000000	0.0000000	0.0000000
phq9_4	0.0000000	0.3089800	0.0000000	0.0000000	0.1740729	0.0000000	0.2037225	0.0000000
phq9_5	0.1596752	0.0000000	0.0000000	0.1740729	0.0000000	0.0000000	0.0000000	0.0000000
phq9_6	0.0000000	0.2057773	0.0000000	0.0000000	0.0000000	0.0000000	0.0000000	0.1622169
phq9_7	0.0000000	0.0000000	0.0000000	0.2037225	0.0000000	0.0000000	0.0000000	0.2162233
phq9_8	0.0000000	0.0000000	0.0000000	0.0000000	0.0000000	0.1622169	0.2162233	0.0000000

Regularized GGM network (glasso; qgraph)

	phq9_1	phq9_2	phq9_3	phq9_4	phq9_5	phq9_6	phq9_7	phq9_8
phq9_1	0.0000000	0.2754253	0.0000000	0.0000000	0.1268758	0.0337540	0.0000000	0.04036919
phq9_2	0.2754253	0.0000000	0.1310087	0.2651232	0.0000000	0.1895486	0.0986628	0.05424744
phq9_3	0.0000000	0.1310087	0.0000000	0.1302180	0.0845758	0.1097117	0.1042531	0.00000000
phq9_4	0.0000000	0.2651232	0.1302180	0.0000000	0.1477334	0.0526319	0.1661313	0.00000000
phq9_5	0.1268758	0.0000000	0.0845758	0.1477334	0.0000000	0.1082317	0.0452132	0.00000000
phq9_6	0.0337540	0.1895486	0.1097117	0.0526319	0.1082317	0.0000000	0.0955732	0.10804007
phq9_7	0.0000000	0.0986628	0.1042531	0.1661313	0.0452132	0.0955732	0.0000000	0.13159693
phq9_8	0.0403691	0.0542474	0.0000000	0.0000000	0.0000000	0.1080400	0.1315969	0.00000000
